# Normative data of laser evoked potentials using Nd:YAP laser

**DOI:** 10.1016/j.cnp.2026.10.002

**Published:** 2026-10-06

**Authors:** Egle Sukockiene, Caroline Perchet, Christelle Créac'h, Luis Garcia-Larrea, Agustina M. Lascano

**Affiliations:** a Division of Neurology, Department of Clinical Neuroscience, Geneva University Hospitals and University of Geneva, Faculty of Medicine, Geneva, Switzerland; b Central Integration of Pain (NeuroPain) Lab-Lyon Neuroscience Research Centre, INSERM U1028, CNRS, UMR5292, Université Claude Bernard, Bron, France; c Centre d'Evaluation et Traitement de la Douleur du Centre Hospitalier Universitaire de Saint-Etienne, Saint-Etienne, France

**Keywords:** Nociception, Spinothalamic tract, Pain, Somatosensory, Central nervous system

## Abstract

**Objectives:**

To establish a robust multi-centre normative database for Nd:YAP laser-evoked potentials (LEPs) across distal and proximal stimulation sites, and to quantify the impact of laser spot size (energy density) and demographic factors on LEP parameters.

**Methods:**

One hundred nine healthy adults (57 females) were recruited from three centres (Geneva, Lyon, Saint-Etienne). Nd:YAP laser pulses were delivered with a 3-mm beam (Centre 1) or 4-mm beam (Centres 2–3). LEPs were recorded from standard EEG sites (including Fz/Cz/Pz and contralateral temporal leads). Stimulation sites included hand dorsum (*n* = 109), foot dorsum (*n* = 80), thigh and upper neck (both *n* = 43). N1, N2, and P2 latencies and amplitudes were measured and normal limits estimated as mean ± 99.9% confidence interval. ANCOVA (age-corrected) tested spot-size effects; repeated-measures ANOVA tested site effects; correlations assessed age/height associations.

**Results:**

The vertex N2–P2 complex was consistently recorded across centres and sites (100% for hand, thigh, neck; ∼97% for foot). N1 detection was less consistent, particularly with larger spot size and at the foot. Smaller spot size (higher energy density) yielded shorter N2/P2 latencies and larger N2–P2 amplitudes for hand stimulation, and shorter P2 latency for foot stimulation. Sex and height showed no significant effects. Increasing age was associated with longer N2/P2 latencies and reduced N2–P2 amplitudes for distal sites, with minimal age effects at proximal sites.

**Conclusions:**

This study provides the most comprehensive Nd:YAP LEP normative dataset to date, including distal and proximal sites and spot-size–specific reference limits.

**Significance:**

These data support standardized, high-specificity clinical interpretation of LEPs for assessing small-fibre and spinothalamic pathway dysfunction.

## Highlights

• Laser-evoked potentials provide a robust window into human nociceptive pathways.
• A small variation in laser spot (3 versus 4 mm) alters LEP latencies and amplitudes.
• Multi-centre norms enable reliable LEP interpretation across laboratories.

## 1 Introduction

The International Federation of Clinical Neurophysiology (IFCN) and the International Association for the Study of pain (Neuropathic SIG-IASP) have recognized laser evoked potentials (LEPs) as a reliable neurophysiological method for exploring the nociceptive pathways and assessing neuropathic pain (Cruccu et al., 2008; Finnerup et al., 2016). Laser pulses specifically activate thinly-myelinated Aδ and amyelinic C fibres, without activating large myelinated Aβ fibres (Treede, 2003). Intracortical correlates of LEP components, identified through high-density EEG source analysis and validated by intracranial EEG studies, have revealed the key brain regions contributing to the scalp-recorded LEPs, all of them pertaining to the pain matrix, and including the posterior insula and medial parietal operculum, the S1 and S2 cortices, anterior insula, mid-anterior cingulate, supplementary motor area and possibly dorsolateral prefrontal areas (Bradley et al., 2017; Garcia-Larrea et al., 2003; Kakigi et al., 2005; Ploner et al., 2002; Ploner et al., 1999; Vogel et al., 2003).

LEPs have provided significant data on the characterization of painful neuropathies (Di Stefano et al., 2017; Gendre et al., 2024; Kakigi et al., 1991; Mouraux et al., 2012; Truini et al., 2009), the distinction between neuropathic versus non-neuropathic pain(Leone et al., 2023; Lorenz et al., 1996) and the mechanistic dissection of neuropathic syndromes (Garcia-Larrea et al., 2002; Truini and Cruccu, 2016; Truini et al., 2013) including the pathophysiological distinction of constant versus paroxysmal pain (Truini et al., 2010; Truini et al., 2013). LEPs allowed for diagnosis of previously unrecognized central pain syndromes (Convers et al., 2020; Garcia-Larrea et al., 2010) and demonstrated nociceptive disinhibition in migraine and nociplastic pain (de Tommaso, 2005; Valeriani et al., 2003; Van Assche et al., 2020). However, despite these relevant findings and other methodological advantages of LEPs, their use in routine clinical practice remains limited and subject to ongoing debate (Garcia-Larrea and André-Obadia, 2025; Truini et al., 2023). The lack of sufficient data on sensitivity and specificity for specific disorders, along with absence of international consensus guidelines on recording, interpretation, and normative values, has led to only moderate recommendation for their use in the clinics (Truini et al., 2023).

While solid state, particularly Nd:YAP lasers, are now more widespread than gas-based CO_2_ laser systems, normal data are available mostly for CO2 laser-evoked responses (de Tommaso et al., 2020). Normative LEP on solid-state Nd:YAP data remain limited, have not been reported for regions beyond the face and limbs (Di Stefano et al., 2017); and have not been compared across laboratories using the same equipment but partially dissimilar procedures. This appears particularly important since a recent consensus report concluded that the variability due to age, sex and height factors defied normalisation and requested single-laboratory normative ranges (de Tommaso et al., 2020). Our objectives were therefore to gather data from different laboratories using the same laser Nd:YAP stimulus but diverse procedures in terms of energy density, and establish a comprehensive normative database including responses to stimulation of distal (hands, feet) and proximal body regions (thigh and neck). The data presented here were derived from three centres using the same type of laser equipment and aim to provide clinicians with robust and clinically relevant reference values.

## 2 Methods

### 2.1 Study population

A total of 109 healthy adult volunteers of both sexes (57 females), aged 20–76 years, were recruited from the Neurology Department at University Hospitals of Geneva, Switzerland (Centre 1, *n* = 37), the Centre for Neuroscience (CRNL), INSERM & Claude Bernard Lyon University, France (Centre 2, *n* = 29), and the University Hospital of Saint-Etienne, France (Centre 3, *n* = 43). Eligibility criteria included the absence of neurological, psychiatric, or chronic pain disorders, as well as the absence of psychotropic medication. Pregnancy and breastfeeding were specified as exclusion criteria. All participants provided informed consent, and the study received approval from the relevant local Ethics Committees.

There was no significant difference in the proportion of men and women but there was a significant difference in mean age across centres (F(2,106) = 10.95; <0.001), subjects from Centre 3 being significantly older in the average (46,8 ± 17,1 years) than those in Centre 1 (35 ± 7,7 years; t(106) = 3.97; *p* < 0.001) and Centre 2 (34,2 ± 12,1, t(106) = −3.94; *p* < 0.001). The effect of age was therefore controlled for in subsequent comparative analyses.

### 2.2 Stimulation

Heat pulses of 5 ms duration were generated by an infrared Nd:YAP laser unit (Yttrium Aluminium Perovskite; 1.34 um wavelength; ElEn, Florence, Italy) and guided by a 10-m fibre-optic cable. The diameter of the laser beam, and hence of the stimulated skin spot, was 3 mm in Centre 1, and 4 mm in Centres 2 and 3, resulting in different levels of energy density, respectively 14.14 J/cm^2^ and 7.96 J/cm^2^ for a stimulation at 1 J. Stimuli were applied in all cases with an interstimulus interval of 10–15 s. A visible laser red beam was used to visually outline the stimulated area. Before recordings, the stimulus intensity was adjusted to induce a mild painful pinprick sensation, just above the nociceptive threshold, often described as “pulling a hair”, or “receiving a drop of boiling water”.

To prevent both habituation and nociceptor sensitization, the laser beam was slightly repositioned after each stimulation. Participants were instructed to maintain focus throughout LEP testing by mentally counting the stimuli or rating pain on a 10-point numerical scale, which was recorded at the end of the procedure. Both the participants and the experimenters wore protective goggles during the sessions.

Four body areas were stimulated:

• The dorsum of the hand (C6, superficial radial nerve) was stimulated in all Centres (109 subjects).
• The dorsum of the foot (L5, superficial fibular nerve) was stimulated in 80 subjects from Centres 1 and 3.
• The anterior part of the thigh (L3, anterior femoral cutaneous nerve) and upper neck (C3, cervical plexus) were stimulated in Centre 3 (43 subjects).

### 2.3 Recording parameters

EEG signals were amplified, sampled at 200–512 Hz and band-passed filtered at 0.2–70 Hz (Centre 1) or 0.3–30 Hz (Centres 2 and 3). Eye movements (saccades and blinking) were monitored continuously in all Centres and trials discarded when ocular artifacts impacted on the analysis window. Although the total number of electrodes varied among Centres, (*n* = 8 in Centre 1; *n* = 32 in Centre 2; *n* = 20 in Centre 3), all montages included at least Fz, Cz, Pz of the 10–20 International System, referenced either to the nose or linked earlobes, along with at least one electrode positioned in the temporal region contralateral to the stimulation (T7, T8). Data for analysis were taken from these sites.

Averaging included 20–30 trials per stimulation site, either performed automatically by the manufacturer's software or completed offline following, epoching and filtering of the continuous EEG. The selected epochs covered 900 ms post-stimulus, with an additional 100 ms pre-stimulus baseline.

### 2.4 LEP components

We analysed the three primary scalp components observed within a 500 ms post-stimulus window, commonly referred to as “N1”, “N2”, and “P2”. These components were identified based on their scalp topography and temporal sequence (Fig. 1). The N1 is the earliest component, with latency varying from 100 ms to 200 ms depending on the region stimulated, appearing as a small negative-going response (‘N1’) distributed over the temporal scalp and showing a phase reversal (‘P1’) at midline frontal and frontopolar electrodes. This lateralised response is followed by a negative-positive “vertex complex” with maximal amplitude over the midline, composed by the N2 component (negative wave peaking at 150–300 ms) followed by a large positive wave (P2) peaking between 250 and 500 ms in healthy individuals. Fig. 1 illustrates the different LEP components elicited by stimulation of the left-hand dorsum.

**Fig. 1:**
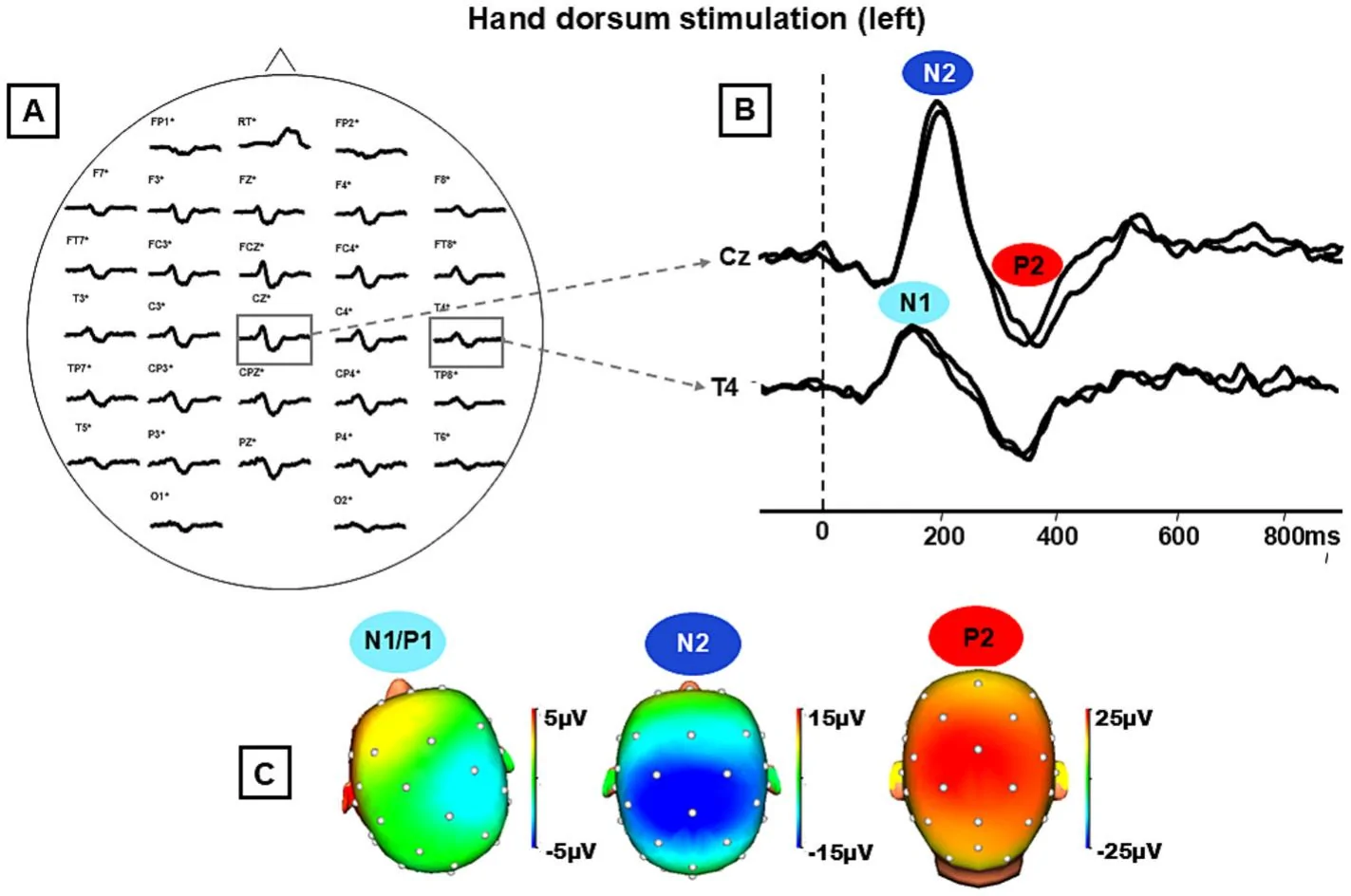
Time-course and topographic maps of laser-evoked potentials following hand dorsum stimulation. A. LEPs recorded with a 32-electrode montage. B. Vertex (Cz) and lateral temporal (T4) LEPs to left hand stimulation, illustrating the morphology and reproducibility of the main components analysed in this work. C. Topographic colour maps illustrating the scalp distribution of the 3 main components: the N1 (pale blue) is distributed over the lateral scalp contralateral to stimulation, with its phase reversal P1 (yellow) largely distributed over the ipsilateral frontal and frontopolar scalp. The N2 and P2 are largely distributed over the scalp but maximal over the midline. (For interpretation of the references to colour in this figure legend, the reader is referred to the web version of this article.)

Latencies of N1, N2 and P2 were measured from stimulus onset. Amplitudes were calculated from baseline to peak for N1 and as peak-to-peak amplitude for N2-P2 complex. Absolute values of side-to-side latency and amplitude asymmetry were used to derive normal limits for each component after the 4 sites of stimulation, as well as inter-side differences for hand and foot LEPs. Data from right and left side stimulation were averaged to assess potential effects of age, height and sex of participants.

### 2.5 Statistical analyses

Descriptive statistics included the mean, standard deviation (SD), hit detection rate, and estimated normal limits based on the 99.9% confidence interval (CI) for each component.

Since subjects could be stimulated via laser beams of different diameters leading to dissimilar energy densities (3 mm in Centre 1; 4 mm in Centres 2 and 3), a comparison of LEP results to different stimulus diameter was performed using ANCOVA, corrected for age. The effect of stimulated body region on LEP latencies and amplitudes was studied in a subset of *n* = 43 subjects who received stimulation at 4 sites (hand, foot, thigh and neck) via repeated measures ANOVA, with stimulated area as within-subject factor. Post-hoc analyses were performed with Tukey's Honestly Significant Difference (HSD) test after factorial ANOVA or ANCOVA, and with Bonferroni test following repeated measures ANOVA.

Latencies and amplitudes to right and left side stimulation were compared via paired *t*-tests or Wilcoxon signed-rank tests as appropriate. Asymmetry indices were calculated based on the absolute differences between right and left sides. Normal limits were defined as the upper asymmetry limit (mean + 3SD).

Pearson product-moment or Spearman correlations were used to study potential correlations between age, height and LEP latencies and amplitudes.

## 3 Results

Descriptive values for LEPs elicited by stimulation of the hand, foot, neck, and thigh, together with their corresponding normal limits (mean ± 99.9% CI), are summarized in Table 1. Normal limits are described separately for age (before / after 55 years), and for stimuli using 3 mm and 4 mm laser beam diameters (spot size), since significant differences were obtained when changing spot size (see below). Fig. 2 shows laser beam diameter-dependent differences in N2 and P2 latency and amplitude, displayed as rainbow plots for 3 mm and 4 mm stimulation of the hand and foot dorsum.

**Table 1:** Normative values of Laser-Evoked Potentials (LEPs).

	Spot size	Energy density (mJ/mm^2^)	Age (y)	Hit Rate	N1					
					N1 Latency (ms)				N1 amplitude (uv)	
					Mean + 1.96 SD	FPR (%)	Mean + 3 SD	FPR (%)	Median - 99% quartile	FPR (%)
	**3 mm**	**14.14**	**<55**	36/36 (100%)	180	2.8	190	0.0	1.12	2.8
**Hand**			**>55**	NA	NA	NA	NA	NA	NA	NA
	**4 mm**	**7.96**	**<55**	48/54 (88.8%)	204	2.6	225	0.0	1.79	2.6
			**>55**	18/18 (100%)	223	5.9	249	0.0	1.06	5.9
	**3 mm**	**14.14**	**<55**	34/35 (97.1%)	220	0.0	231	0.0	0.30	2.9
**Foot**			**>55**	NA	NA	NA	NA	NA	NA	NA
	**4 mm**	**7.96**	**<55**	14/28 (50%)	250	0.0	272	0.0	1.17	7.1
			**>55**	8/15 (53.3%)	261	0.0	284	0.0	2.11	12.5
**Thigh**	**4** **mm**	**7.96**	**<55**	21/28 (75%)	203	4.8	220	0.0	1.32	4.8
			**>55**	8/15 (53.3%)	221	0.0	246	0.0	1.54	12.5
**C3 dermatome**	**4** **mm**	**7.96**	**<55**	23/28 (82%)	184	4.3	207	4.3	2.01	4.3
			**>55**	13/15 (86.6%)	186	0.0	210	0.0	0.78	7.1


				Hit Rate	**Vertex complex**									
	Spot size	Energy density (mJ/mm^2^)	Age (y)		N2 Latency (ms)				P2 Latency (ms)				N2P2 Amplitude (μV)	
					Mean + 1.96 SD	FPR (%)	Mean + 3 SD	FPR (%)	Mean + 1.96 SD	FPR (%)	Mean + 3 SD	FPR (%)	Median - 99% quartile	FPR (%)
	**3 mm**	**14.14**	**<55**	36/36 (100%)	225	0.0	237	0.0	333	0.0	350	0.0	9.78	2.8
**Hand**			**>55**	NA	NA	NA	NA	NA	NA	NA	NA	NA	NA	NA
	**4 mm**	**7.96**	**<55**	54/54 (100%)	252	5.6	274	0.0	401	3.7	443	0.0	6.20	1.9
			**>55**	18/18 (100%)	303	5.6	341	0.0	441	5.6	490	0.0	7.82	5.6
	**3 mm**	**14.14**	**<55**	35/35(100%)	275	0.0	290	0.0	386	0.0	403	0.0	5.75	2.8
**Foot**			**>55**	NA	NA	NA	NA	NA	NA	NA	NA	NA	NA	NA
	**4 mm**	**7.96**	**<55**	28/28 (100%)	250	0.0	272	0.0	315	3.6	346	0.0	4.91	3.6
			**>55**	13/15 (86.6%)	344	0.0	378	0.0	500	0.0	549	0.0	2.06	7.7
**Thigh**	**4** **mm**	**7.96**	**<55**	28/28 (100%)	270	3.6	295	0.0	401	0.0	437	0.0	6.25	0.0
			**>55**	15/15 (100%)	254	0.0	265	0.0	395	6.7	413	0.0	4.69	6.7
**C3 dermatome**	**4** **mm**	**7.96**	**<55**	28/28 (100%)	213	3.6	231	0.0	386	3.6	430	3.6	4.86	3.6
			**>55**	15/15 (100%)	225	6.7	244	0.0	410	13.3	458	0.0	5.51	6.7

NA = not available; SD = standard deviation; CI = confidence Interval; FPR = False positive rate.

**Fig. 2:**
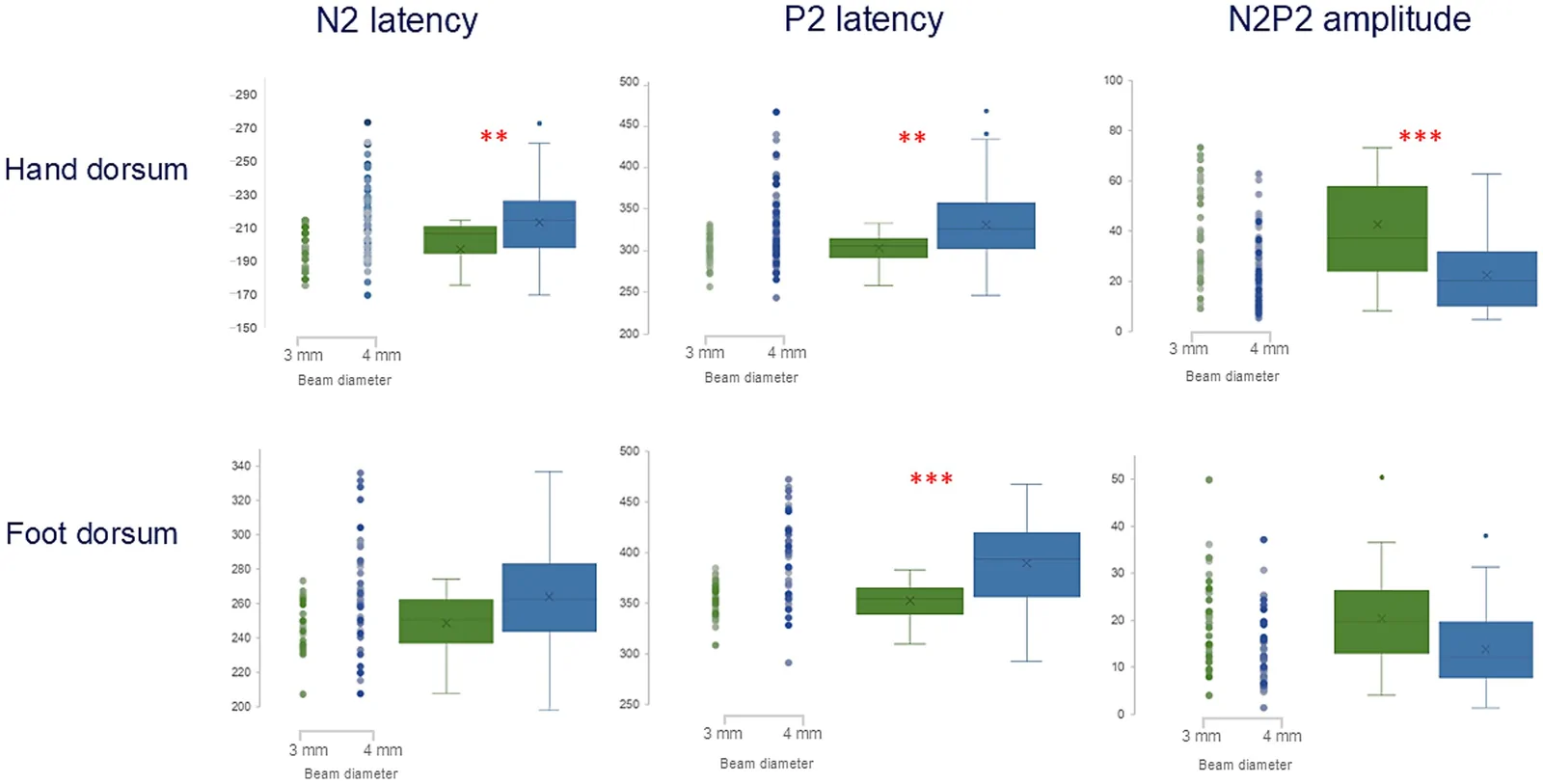
Laser beam diameter-dependent variations in N2 and P2 latency and amplitude. Rainbow and whisker plots illustrate differences in response latencies and amplitudes according to beam diameter (3 mm vs 4 mm). Results obtained with 3 mm stimulation are shown in green; those obtained with 4 mm stimulation in blue. A greater dispersion of N2 and P2 latencies and lower amplitude is observed with 4 mm laser-beam (blue) following both hand (top panel) and foot dorsum (bottom panel) stimulation. Statistical significance is indicated as ** *p* < 0.05 and *** *p* < 0.01. (For interpretation of the references to colour in this figure legend, the reader is referred to the web version of this article.)

The vertex complex (N2 and P2 components) was consistently obtained across all Centres and stimulation sites, except for 2/80 participants following foot stimulation (Center 1 and 3). A lateralized N1 component following hand stimulation was detected in all participants in Center 1, in 93% of cases in Center 2 and in 91% in Center 3. Following foot stimulation performed in Center 1 and 3 only, the N1 was detected in 97% of cases in Center 1 and in 52% in Center 3.

Stimulation of the thigh and the lateral neck was performed in Centre 3 only: the vertex complex N2-P2 was identified in 100% of cases and the lateralized N1 in 67.4% and 84% respectively for thigh and neck.

Analysis of covariance (ANCOVA) on the effect of laser beam diameter (spot size) was performed for hand and foot stimulation (neck and thigh LEPs were all obtained with a 4 mm spot diameter). Analyses were corrected for age (ANCOVA with spot size as fixed factor and age as covariate) and showed significant differences in the midline response (N2–P2), with shorter latencies and higher amplitudes in LEPs obtained with a higher density via a smaller spot size. Specifically, for **hand stimulation**, differences corrected for age were significant for N2 latencies (205 ± 11.4 ms for 3 mm vs. 215 ± 26.01 ms for 4 mm spots; *F*(1,106) = 5.41; *p* = 0.002), and P2 latencies (304 ± 16.5 ms vs. 327 ± 42.91 ms; *F*(1,106) = 10.33; *p* < 0.002), as well as for N2P2 amplitude (40.5 ± 25.4 uV vs. 24.6 ± 13.4 uV; *F*(1,106) = 21.2; *p* < 0.001). For **foot stimulation,** conversely, significant differences concerned exclusively P2 latencies (358 ± 16.6 ms for 3 mm vs. 387 ± 41.47 ms for 4 mm; *F*(1,75) = 15.56; *p* < 0.001). Neither N2 latency nor N2P2 amplitude showed significant differences linked to spot size for foot stimulation (*F*(1,75) = 0.56) and *F*(1,75) = 3.58 respectively). No significant effect was observed regarding the early lateralised N1 after hand or foot stimuli.

### 3.1 Side-to-side asymmetry (Table 2)

Mann–Whitney U testing revealed no significant differences between right and left stimulation in Center 1 for any outcome measure, including N1 latency (*p* = 0.20), N1 amplitude (*p* = 0.41), N2 latency (*p* = 0.42), P2 latency (*p* = 0.37), and N2–P2 amplitude (*p* = 0.36). Asymmetry indexes were computed for hand and foot, using absolute differences of latency and amplitude for responses to stimulation of right and left sides (Table 2**)**. The upper limits of normal asymmetry indexes were set at 3 SD over the mean absolute inter-side difference.

**Table 2:** Asymmetry Indexes for Hand and Foot Stimulation (3 mm spot size).

Hand dorsum				
Component	Left Mean +/− SD	Right Mean +/− SD	Absolute ID +/− SD	Upper normal asymmetry limit (mean + 3SD)
N1 latency (ms)	162.49 +/− 8.83	164.37 +/− 10.21	5.96 +/− 6.27	24.76 ms
N1 amplitude (μv)	5.02 +/− 3.41	5.22 +/− 3.67	2.94 +/− 2.25	9.69 μv
N2 latency (ms)	202.50 +/− 12.44	203.04 +/− 10.84	3.70 +/− 5.34	19.72 ms
P2 latency (ms)	299.18 +/− 18.42	301.52 +/− 17.44	11.08 +/− 10.78	43.42 ms
N2P2 amplitude (μv)	41.92 +/− 25.21	44.27 +/− 25.96	9.23 +/− 6.83	29.72 μv


Foot dorsum				
Component	Left Mean +/− SD	Right Mean +/− SD	Absolute ID	Upper normal asymmetry limit (mean + 3SD)
N1 latency (ms)	199.20 +/− 12.61	196.72 +/− 12.91	6.36 +/− 8.41	31.58 ms
N1 amplitude (μv)	2.90 +/− 2.28	2.81 +/− 2.25	1.98 +/− 1.73	7.18 μv
N2 latency (ms)	247.36 +/− 14.08	248.63 +/− 15.32	6.75 +/− 7.13	28.13 ms
P2 latency (ms)	352.28 +/− 17.54	353.99 +/− 18.71	12.46 +/− 9.38	40.59 ms
N2P2 amplitude (μv)	19.59 +/− 9.77	20.77 +/− 10.11	4.79 +/− 4.99	19.76 μv

ID = interside difference; SD = standard deviation; CI = confidence interval; NA = not applicable.

Note that very large side-to-side differences, exceeding 75%, are required to consider as abnormal an amplitude difference between sides.

### 3.2 Influence of sex and height

A two-way between-subjects ANOVA was performed with sex (M/F) and spot diameter (3 mm vs. 4 mm) as fixed factors, for hand and foot LEPs. No effect of sex and latencies or amplitudes, and no spot size * sex interaction was obtained for hand or foot LEPs. In accordance, independent-samples *t*-test, pooling data across all 4 stimulation sites revealed no statistically significant differences in N2 latency (*t*(107) = −1.2; *p* = 0.11), P2 latency (*t(107)* = −1.02; *p* = 0.15), or N2–P2 amplitude (*t(107)* = 0.89; *p* = 0.18) across genders.

No significant correlation was observed between subjects' height and any of the LEP variables examined (N1, N2, P2 latencies, N2P2 amplitude) at any of the stimulation sites.

### 3.3 Influence of age (Fig. 3)

N2 latency to hand and foot stimulation increased significantly with age, for both the 3 mm (hand: *r* = 0.297, *p* = 0.037; foot: *r* = 0.508, *p* = 0.001) and the 4 mm laser beams (hand: *r* = 0.36, *p* = 0.001; foot: *r* = 0.552, *p* < 0.001) as illustrated in Fig. 3 (panels A and D). No significant correlations with age were observed for stimulation at proximal sites, thigh (*r* = 0.029, *p* = 0.854) or upper neck (*r* = 0.298, *p* = 0.520).

**Fig. 3:**
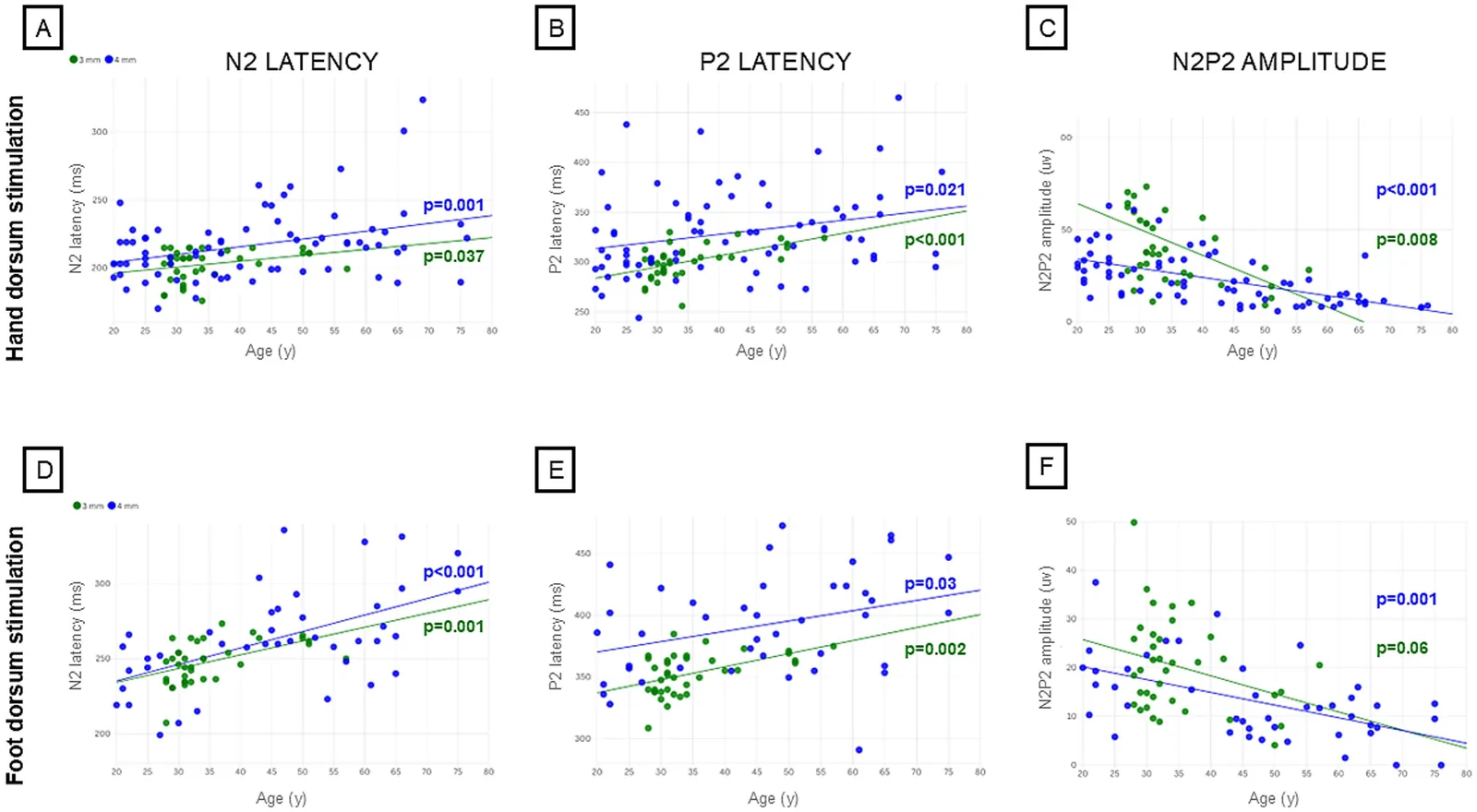
Comparison of 3 mm vs. 4 mm laser diameter stimulation results across age groups. Scatter plots illustrate the distribution of N2 latency, P2 latency, and N2–P2 amplitude ratio following laser stimulation of the hand and foot dorsum using either a 3 mm beam (Centre 1, shown in green) or a 4 mm beam (Centres 2 and 3, shown in blue). After hand stimulation (panels A and B) and foot stimulation (panels D and E), both N2 and P2 latencies increased significantly with age, independently of beam diameter. An age-related decrease in the N2–P2 amplitude ratio was observed following hand dorsum stimulation regardless of beam diameter (panel C) and following foot dorsum stimulation with the 4 mm beam (panel F). (For interpretation of the references to colour in this figure legend, the reader is referred to the web version of this article.)

P2 latency to hand and foot stimulation followed the same trend and increased significantly with age both using the 3 mm beam, (hand: *r* = 0.527, *p* < 0.001; foot: *r* = 0.49, *p =* 0.002), and the 4 mm beam (hand: *r* = 0.271, *p =* 0.021; foot: *r* = 0.332, *p =* 0.03) (Fig. 3, panels B and E). No significant correlations with age were observed for stimulation at the thigh (*r* = 0.585; *p* = 0.08) or upper neck (*r* = 0.706; *p =* 0.06).

N2P2 amplitude decreased significantly with age following hand stimulation with both the 3 mm beam (*r* = −0.426, *p* = 0.008), and the 4 mm beam (*r* = −0.593, *p* < 0.001), while for the foot stimulation a significant negative amplitude correlation emerged only with 4 mm beam (*r* = −0.483, *p* = 0.001) and not with 3 mm beam (*r* = −0.303, *p* = 0.06) (Fig. 3, panel C and F). No significant age-related effects were found at the thigh (*r* = −0.267, *p* = 0.084) or neck (*r* = −0.293, *p* = 0.057)

While decreasing LEP amplitudes with age were evident for hand and foot LEPs (Fig. 3, panel C and F), the correlations with latencies, although significant, showed very small slopes after hand stimulation between 20 and 55–60 years.

### 3.4 Influence of the stimulated areas (Fig. 4)

*N* = 43 subjects received laser stimulation on four sites, two proximal (neck and thigh) and two distal (hand and foot). The effect of site on latencies and amplitudes was assessed with a mixed-design ANOVA with site of stimulation as repeated measure and sex (M/F) as between-factor. A highly significant effect was evident on the latencies of each component (N1: F(3,51) = 45.9; *p* < 0.001); N2: F(3,117) = 143; *p* < 0.001; and P2 (F(3,117) = 708; *p* < 0.001), latencies being shortest to stimulation of the neck, and longest to stimulation of the foot. (Fig. 4).

**Fig. 4:**
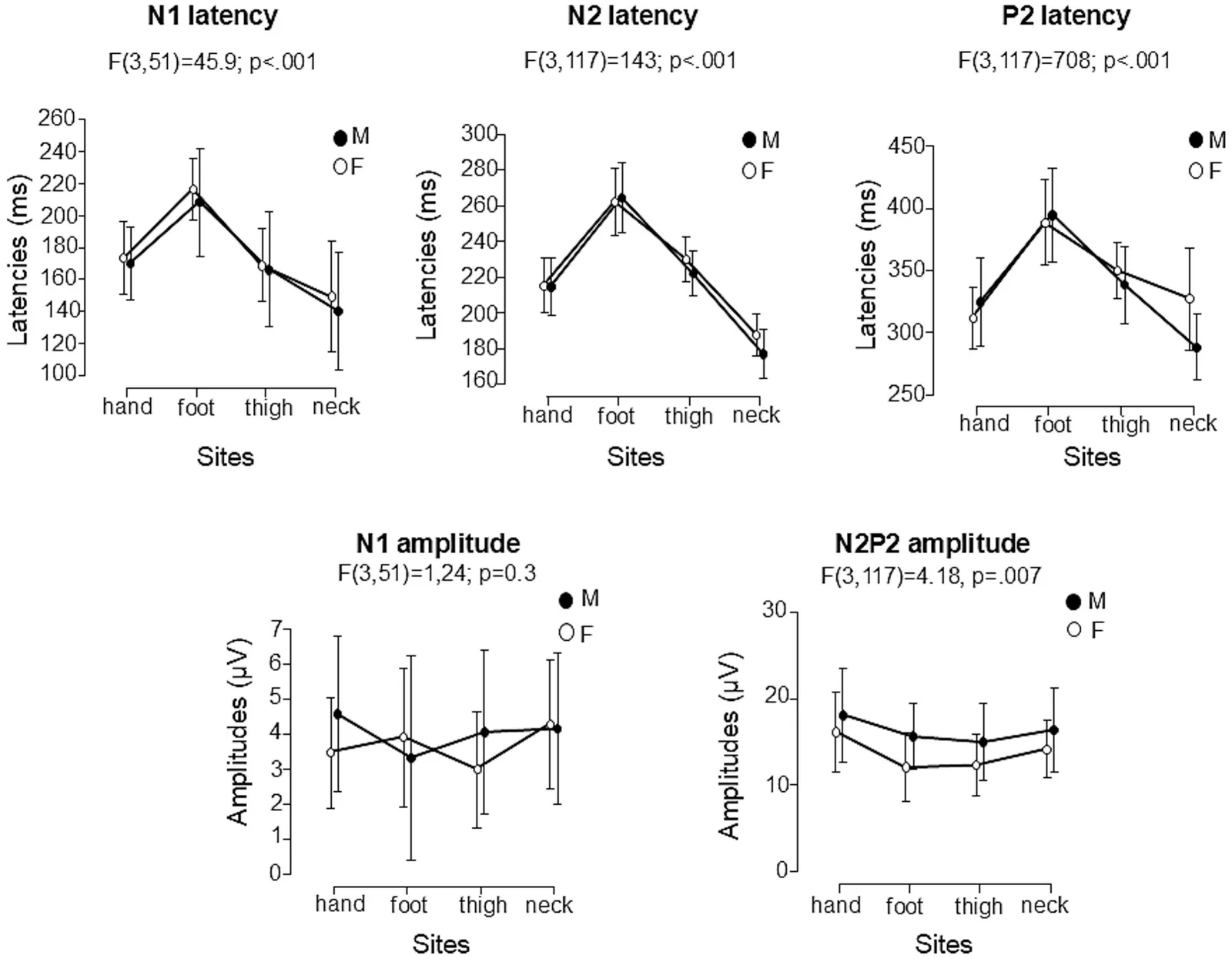
Latency and amplitude of laser-evoked potential components across stimulation sites. Site-dependent effects on LEP latencies and amplitudes were assessed in 43 subjects from Centre 3 who underwent stimulation at four sites (neck, thigh, hand, and foot). The top panel shows N1, N2, and P2 latencies, and the bottom panel displays N1 and N2–P2 peak-to-peak amplitudes. Males are shown as black dots and females as white dots.

A smaller effect on amplitude was also observed (F(3,117) = 4.18, *p* = 0.007), mean N2P2 amplitudes being higher after stimulation of the hand relative to all other sites, which did not differ between them (see Fig. 4). There was no significant effect of sex on latency (N1: F(1,17) = 0.8; *p* = 0.4); N2: F(1,39) = 0.5; *p* = 0.5; and P2 (F(1,39) = 0.8; *p* = 0.4) or amplitude values (N1: F(1,17) = 0.8; *p* = 0.4); N2P2: F(1,39) = 1.8, *p* = 0.2), and no interaction between sex and stimulation site, except for the latency of P2 (F(3,117) = 6.84, *p* < 0.001), explained by the fact that males had longer latencies than females at proximal stimulation sites (neck and thigh) while females had longer latencies for distal stimuli.

## 4 Discussion

This study presents the most comprehensive normative database to date on LEPs to Nd:YAP laser stimulation. It includes data from four distinct stimulation sites, two distal (hand and foot); and two proximal (thigh and neck) and compares two different laser beam sizes (3 mm vs. 4 mm diameter). We believe these features increase the robustness and clinical applicability of the normative data for evaluating patients with suspected lesions or diseases involving thin fibres or spinothalamic pathways, which are critical elements in the diagnosis of neuropathic pain. Deviations from normal ranges, including in asymmetry indexes, may indicate dysfunction in either peripheral thin fibres (focal or diffuse small-fibre neuropathies) or the central spino-thalamo-cortical pathway (Di Stefano et al., 2017; Landmann et al., 2017; Treede, 2003; Truini et al., 2003). Establishing normative values for both distal and proximal sites (e.g., thigh and cervical region) may help confirm or exclude a proximal-distal gradient in length-dependent small-fibre neuropathy, and differential involvement of distal and proximal sites may help in localizing lesions within central pain pathways*.* In this study we tried to emphasize the specificity of LEP abnormalities, by defining abnormal values as 3 SD over/below the mean, and by reporting the corresponding 99.9% confidence intervals (CI). The advantage of this approach is a higher specificity at the expense of sensitivity (Wold et al., 2022). This is particularly appropriate when the aim is not screening but rather the confident identification of clinically meaningful abnormalities. We also note methodological differences with previous publications regarding the influence of height and sex on LEP parameters (de Tommaso et al., 2020). These data are offered as a reference framework, not a rigid standard, leaving full scope for laboratories to define norms that best reflect their populations and technical environments.

The advantages of LEPs in the neurophysiological assessment of pain syndromes are well established (Cruccu et al., 2003; Garcia-Larrea et al., 2003; Perchet et al., 2008; Truini et al., 2023). LEPs allow selective exploration of thermo-nociceptive pathways and can be recorded from virtually any skin surface. A recent expert consensus from the European Academy of Neurology (EAN), the European Pain Federation (EFIC), and the Neuropathic Pain SIG of the International Association for the Study of Pain (IASP) recognized LEPs as a well-established technique for investigating the nociceptive system in patients with neuropathic pain, which “can detect minute, image-proven lesions within the nociceptive system” (Truini et al., 2023). Despite these strengths, the panel surprisingly issued only a weak recommendation for the routine diagnostic use of LEPs. This conservative position likely reflects the limited evidence that could be assessed, as sensitivity and specificity analyses were based on only three studies relying exclusively on skin biopsy as the reference standard (Di Stefano et al., 2017; Lagerburg et al., 2015). Skin biopsies may yield normal findings in 10–24% of patients with small-fibre neuropathy (Devigili et al., 2019; Fabry et al., 2020; Walk et al., 2007), and can be altered in conditions other than neuropathic pain (Marshall et al., 2025; Nolano et al., 2018). Aware of these limitations, the consensus report emphasized the high specificity of LEPs and supported their role as valuable second-line investigations in the assessment of neuropathic pain (Truini et al., 2023).

### 4.1 Specificity and reliability

In this study, we propose defining LEP normal limits using stringent criteria, based on the 99.9% confidence interval for both amplitudes and latencies. While this may hamper the detection of subtle abnormalities, it should enhance the specificity of the technique and limit false positives. LEPs are best considered as a ‘second line’ test, essential to proceed from a diagnosis of ‘probable’ toward ‘definite’ neuropathic pain (Finnerup et al., 2016); therefore, a high specificity is critical to enhance credibility as a reliable diagnostic test and reduce false positive results which can lead to unnecessary interventions. Stringent cutoff levels have allowed for successful detection of thin fibres lesions in the periphery (Di Stefano et al., 2017; Gendre et al., 2024; Ragé et al., 2011) and spinothalamic damage of the central nervous system (Convers et al., 2020; Fillebeen et al., 2025; Garcia-Larrea et al., 2002; Vartiainen et al., 2016), supporting their role as a second-line confirmatory test. Using more liberal normal limits (e.g. 2 SD over the mean) is not advised except for screening purposes, as it leads to an increase of false positives and results in a weak correlation between LEP results and self-reported clinical symptoms, therefore reducing the LEP's diagnostic impact (Wold et al., 2022).

In terms of reliability, the “vertex complex” N2-P2 proved to be consistently recorded in 100% of the subjects for hand, thigh and neck stimulation, and ∼ 97% after foot stimuli. The reliability of the N2-P2 complex has been often underscored in past reviews (e.g. de Tommaso et al., 2020) and contrasts with the difficulty to obtain dependable recordings of the earlier N1 response. Small amplitude and inconsistent presence of LEP N1 have been reported previously and substantially limit its clinical usefulness(de Tommaso et al., 2020; Di Stefano et al., 2017). In our series, N1 was less consistently recorded than the N2-P2 complex, especially to stimulation of the foot, and when it was obtained no clear peak latency could be often determined with certainty. Interpretation may be improved by combining the lateralised negativity with its phase reversal (‘P1’) which develops over frontal regions, and in this line a bipolar montage (temporal T7 or 78 vs. Fz) has been advocated to enhance amplitude and optimise reliability. However, the variability in the scalp maxima of the ‘P1’, which can vary from vertex to frontopolar regions, as well as the frequent contamination of temporal recordings by muscle activity, make that a reliable assessment of N1/P1 activity can only be safely performed by observing its distribution over the entire scalp via topographic maps (see Fig. 1). As a corollary, and despite the potential interest of such an ‘early’ sensory response for clinical diagnosis, we cannot recommend the use of N1 in routine practice, unless it is supported by topographic map assessment.

### 4.2 Technical and subject influences on LEP parameters

#### 4.2.1 Area of irradiated skin

A different diameter of the stimulated skin spot was used by Centre 1 relative to the other 2 laboratories (3 vs. 4-mm), and therefore a relevant feature of the present work is the direct comparison of LEP responses when applying the same nominal laser energy over different surfaces. Because energy density is inversely proportional to the square of the irradiated spot diameter, a nominal energy of 1 J, commonly used with Nd:YAP lasers, results in energy density of 7.96 J/cm^2^ with a 4-mm beam but 14.14 J/cm^2^ with a 3-mm beam –as the same energy is concentrated on a smaller area. Energy density modulates the LEP response: a smaller beam diameter increases the steepness of the local temperature change, therefore enhancing the synchronous activation of A-delta fibres. In our study, stimulation with a 3 mm beam produced significantly higher N2P2 amplitudes and shorter latencies of vertex N2 and P2 components when stimulating the hand, while the differences were much smaller and restricted to P2 latency for foot stimulation, with no significant amplitude difference (Fig. 2). This suggests that the effect of a higher afferent synchronisation may be somewhat ‘diluted’ with increasing distance from the stimulus point to the brain.

#### 4.2.2 Age, height and sex

We observed a consistent correlation between age and both the amplitude and latency of LEP components, independent of spot diameter (Fig. 3). Amplitude drop with increasing age was conspicuous during the whole life span, whereas latency changes, in particular for hand LEPs, were very small between 15 and 55 years of age, and highly variable thereafter. Previous studies using different types of laser stimuli (CO2, Nd:YAP, Th:YAG) have also described age-related changes in LEP responses across age ranges and at different stimulation sites within the same limb, suggesting a progressive loss of thin fibres with age, especially those with higher conduction velocities (de Tommaso et al., 2017). Creac'h and colleagues suggested that prolonged LEP latencies may result from fibre loss and desynchronisation of the ascending volley (Creac et al., 2015), and an age-related decline in epidermal nerve fibre density has been documented (Gøransson et al., 2004). A recent study, however, did not found correlation between LEP amplitude and small-fibre loss in patients with fibromyalgia (FM) (Vecchio et al., 2022), suggesting that fibre loss in FM may not impact on functional fibres (Van Assche et al. EJP 2020), or that the effect of fibre loss may be compensated in FM patients by a higher cognitive drive to laser stimuli, known to enhance LEP amplitude (Lorenz et al., 1996; Wang et al., 2025).

#### 4.2.3 Limitations

This study has several technical limitations. Even if stimulation intensity was determined with a value just above nociceptive thresholds in the three Centres, the participants' subjective perception of the painful stimuli was not documented. Not all subjects underwent bilateral stimulations, so asymmetry coefficients could be assessed in a subset of 37 participants. Only one of the three Centres (Centre 3) provided data from the 4 sites of the stimulation in the same subjects, which prevented us to conduct analyses combining data from the three study cohorts.

## CRediT authorship contribution statement

**Egle Sukockiene:** Data curation, Formal analysis, Investigation, Writing – original draft. **Caroline Perchet:** Conceptualization, Methodology, Supervision, Writing – original draft. **Christelle Créac'h:** Investigation, Methodology, Writing – review & editing. **Luis Garcia-Larrea:** Conceptualization, Supervision, Writing – review & editing. **Agustina M. Lascano:** Conceptualization, Methodology, Supervision, Writing – original draft, Writing – review & editing.

## Funding

The LEP stimulation device at the Geneva University Hospitals, Switzerland, was financed by the Carlos and Elsie De Reuter Foundation.

## Declaration of competing interest

ES, CC, CP and LGL have no COI to disclose. AML received honoraria for advisory board participation from Argenx, Johnson and Johnson, AMGEN and Roche, consulting from ADC Therapeutics, Biogen, UCB and Alexion, and for presentations from Alexion, UCB, CSL Behring, Takeda and Sanofi. These activities are unrelated to the current manuscript.

## Data Availability

The authors confirm that the data supporting the findings of this study are available upon request.
